# Case Report: Adjuvant Crizotinib Therapy Exerted Favorable Survival Benefit in a Resectable Stage IIIA NSCLC Patient With Novel *LDLR–ROS1* Fusion

**DOI:** 10.3389/fonc.2022.837219

**Published:** 2022-03-01

**Authors:** An-guo Chen, Dong-sheng Chen, Si Li, Le-le Zhao, Ming-zhe Xiao

**Affiliations:** ^1^Department of Thoracic Surgery, The First Affiliated Hospital of Anhui Medical University, Hefei, China; ^2^The Medical Department, Jiangsu Simcere Diagnostics Co., Ltd., Nanjing, China; ^3^Nanjing Simcere Medical Laboratory Science Co., Ltd., Nanjing, China; ^4^The State Key Laboratory of Translational Medicine and Innovative Drug Development, Jiangsu Simcere Diagnostics Co., Ltd., Nanjing, China

**Keywords:** *ROS1* fusion, crizotinib, adjuvant treatment, NSCLC, case report

## Abstract

Novel adjuvant strategies are needed to optimize outcomes after complete surgical resection in patients with early-stage non-small-cell lung cancer (NSCLC). The adjuvant treatment of ROS Proto-Oncogene 1 (*ROS1*) fusion-positive resected NSCLC is challenging because there is no curative confirmed randomized controlled trial. Next-generation sequencing (NGS) and immunohistochemistry (IHC) staining were performed on the biopsy sample. In this case, we identified a novel *LDLR–ROS1* fusion in a resectable stage IIIA NSCLC patient. The patient received crizotinib as adjuvant treatment and achieved recurrence-free survival (RFS) for 29 months, without significant symptoms of toxicity. In this case, we report a novel *LDLR–ROS1* fusion responding to crizotinib in a patient with lung adenocarcinoma, supporting the use of adjuvant treatment with the ROS1 inhibitor exerting clinical survival benefit in ROS1 fusion-positive resected NSCLC.

## Introduction

Novel adjuvant strategies are needed to optimize outcomes after complete surgical resection in patients with early-stage non-small-cell lung cancer (NSCLC). The ADAURA trial and the IMpower010 trial have demonstrated significant clinical benefit in patients with resectable NSCLC who received targeted (1) and immune adjuvant therapy (2), respectively. Herein, we reported that the use of crizotinib, a ROS1 inhibitor, exerted favorable survival benefit (exceeded 29 months) in a resectable stage IIIA NSCLC patient with novel *LDLR–ROS1* fusion.

## Case Report

**Figure 1:**
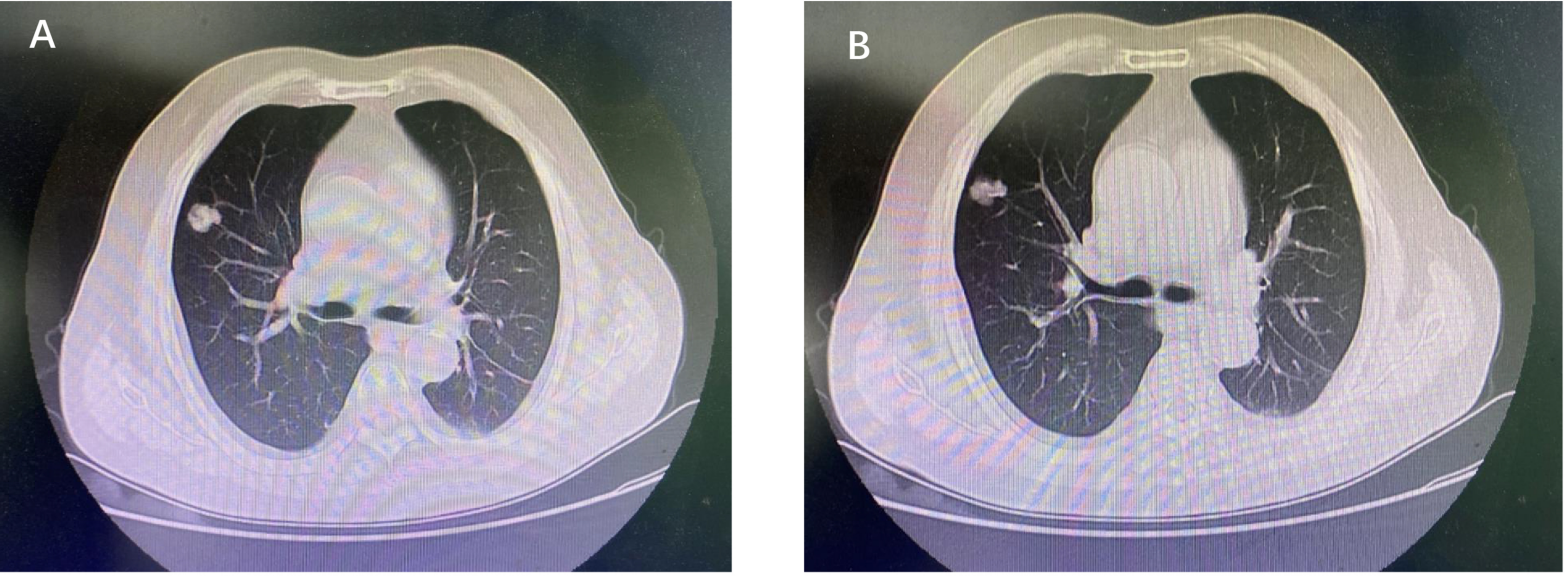
**(A, B)** Computed tomography of chest revealed a space-occupying lesion in the upper lobe of the right lung.

A 72-year-old Chinese male patient was admitted to the first affiliated hospital of Anhui Medical University in June 2019, complaining of irregular cough, having smoked for 40 years. Computed tomography on the chest revealed a space-occupying lesion in the upper lobe of the right lung (**Figures 1A, B****)**. After the relevant preoperative inspection, right lobectomy and lymph node dissection were performed subsequently in June 2019. Postoperative pathological examination revealed complete surgical resection (R0), tumor involvement in the chest wall, and metastases in the positive hilar lymph node (3/4, tumor cells were detected in 3 of the 4 examined hilar lymph nodes), and there were no metastases in the distal lymph nodes. Ultimately, the disease was diagnosed as a stage IIIA lung adenocarcinoma with hilar node metastasis (T3N1M0, according to AJCC 8th edition). Considering the long history of smoking and tumor invasion, postoperative adjuvant therapy was proposed.

**Figure 2:**
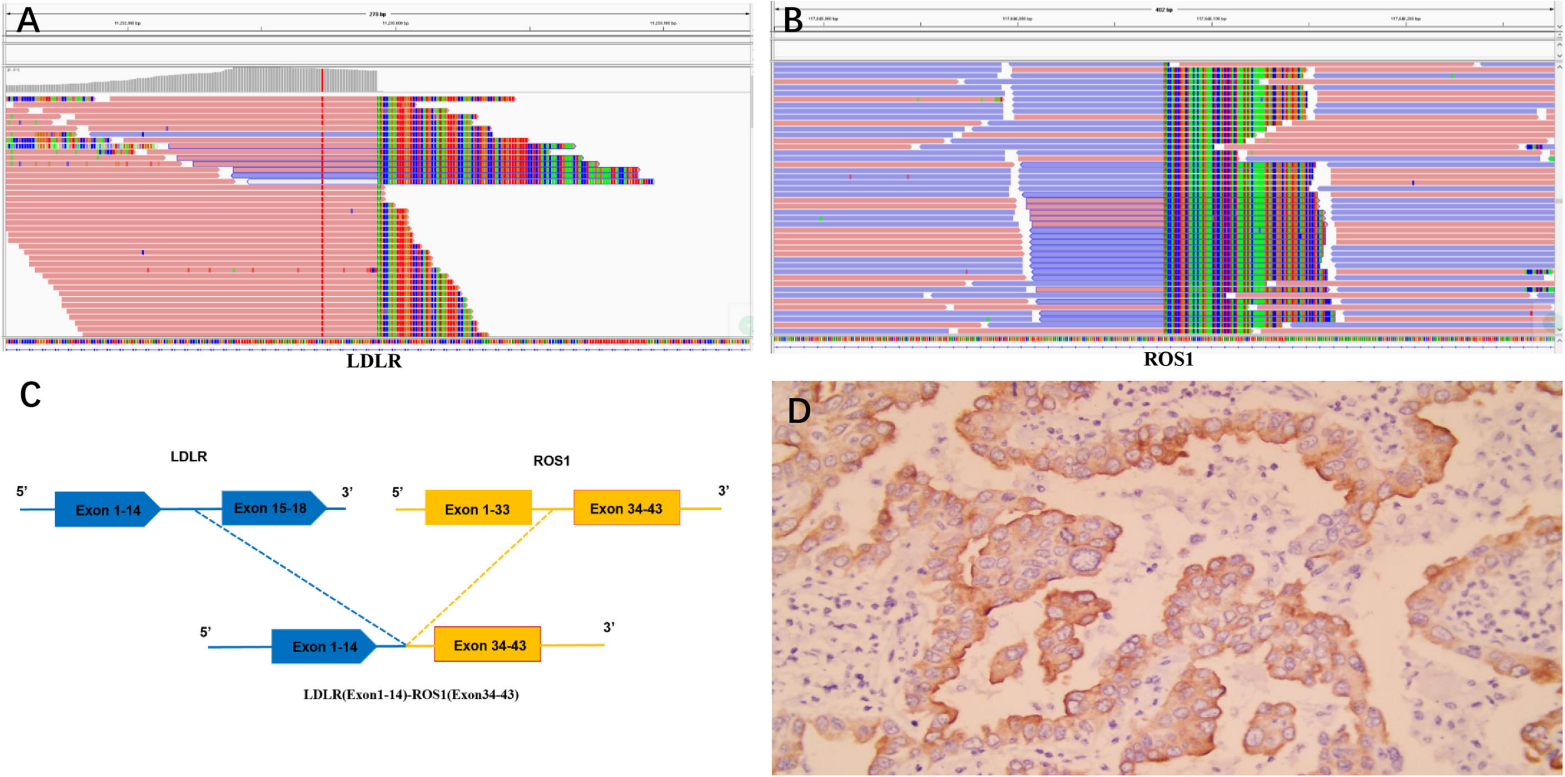
**(A–C)** The Integrative Genomics Viewer snapshot of *LDLR–ROS1* fusion; *LDLR–ROS1* included exons 1–14 of *LDLR* and exons 34–43 of *ROS1*. **(D)** Immunohistochemical staining results of *ROS1* fusion.

**Figure 3:**
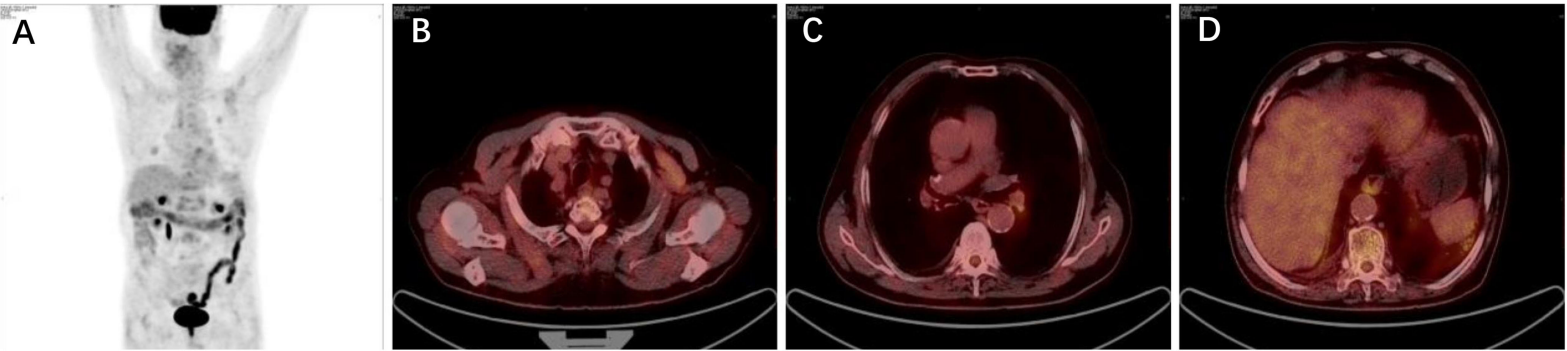
**(A–D)** Clinical and radiological CT of the last follow-up in October 2021.

As the patient explicitly refused further chemotherapy and sought the possibility of adjuvant targeted therapy, next-generation sequencing analysis *via* DNA-based hybrid capture based on a pan-cancer 539-gene panel of the biopsy of primary foci sample was conducted in a CAP-certificated laboratory. A novel *LDLR* (Exon1–14)–*ROS1* (Exon34–43) fusion variant (44.8% abundance) was detected and confirmed by immunohistochemistry (IHC) staining (**Figures 2A–D**). No other key driver gene mutations were detected. Crizotinib (250 mg orally twice daily) was immediately administered beginning in June 2019. Follow-up examinations were performed every 6 months after surgery, clinical and radiological follow-up showed no evidence of progression or recurrence, and the recurrence-free survival (RFS) had exceeded 29 months by the time of submission (December 2021) (**Figures 3A–D**), without significant symptoms of toxicity.

## Discussion

The adjuvant treatment of *ROS1* fusion-positive resected NSCLC is challenging because no confirmed randomized controlled trial has been reported. In this case, we identified a novel *LDLR–ROS1* fusion gene in a lung adenocarcinoma patient. Also, we considered that the patient was sensitive to treatment with the ROS1 inhibitor that the RFS had exceeded 29 months, supporting the use of adjuvant treatment with the ROS1 inhibitor exerting clinical survival benefit in ROS1 fusion-positive resected NSCLC.

*ROS1* has proven to be a valuable therapeutic target in patients with NSCLC; the prevalence of *ROS1* rearrangements is estimated to be 1%–2% of NSCLC patients with at least 20 fusion partners already reported (3–6). It has been established that genomic instability is considered an early step of tumorigenesis, and many fusions are caused by genetic instability. The fusion of *LDLR–ROS1* included exons 1–14 of *LDLR* and exons 34–43 of *ROS1*; this novel *ROS1* fusion retained the complete kinase domain of ROS1 and might lead to abnormal ROS1 activation. Immunohistochemistry results also confirmed the presence of ROS1 fusion.

Both the ADAURA trial and the IMpower010 trial have demonstrated significant clinical benefit in patients with resectable NSCLC who received targeted and immune adjuvant therapy. In the ADAURA trial, the benefit of favoring osimertinib with respect to RFS was observed consistently across all predefined subgroups (1). Moreover, IMpower010 also showed an RFS benefit with atezolizumab versus best supportive care after adjuvant chemotherapy in patients with resected stage II–IIIA NSCLC (2). In our case, postoperative targeted therapy demonstrated a good curative effect, the patient had clinical and radiological follow-up highlighting no evidence of progression or recurrence, and the RFS had exceeded 29 months, which exceeded the median RFS of resectable stage IIIA NSCLC in the placebo group in the ADAURA trial (less than 12 months).

There are also some limitations in our present study. Firstly, this is only a one-patient case report, and more cases are needed to analyze the correlation survival benefit of resectable NSCLC patients with *ROS1* fusion. Secondly, the biological function of *LDLR–ROS1* should be further investigated. All in all, our case report expands the spectrum of *ROS1* fusion types and may recommend a promising option to resectable NSCLC patients with *ROS1* fusion intending to target therapy.

## Data Availability Statement

The datasets presented in this study can be found in online repositories. The names of the repository/repositories and accession number(s) can be found in the article/supplementary material.

## Ethics Statement

Written consent for publication was obtained from the patient.

## Author Contributions

We were all involved in the clinical care and management of the patient, collection of the data, and drafting of the manuscript. D-SC, SL, L-LZ and M-ZX collected the data and wrote the first draft of the manuscript. A-GC supervised the work. All authors contributed to the article and approved the submitted version.

## Conflict of Interest

Authors D-SC, SL, L-LZ and M-ZX were employed by company Simcere Diagnostics Co., Ltd.

The remaining authors declare that the research was conducted in the absence of any commercial or financial relationships that could be construed as a potential conflict of interest.

## Publisher’s Note

All claims expressed in this article are solely those of the authors and do not necessarily represent those of their affiliated organizations, or those of the publisher, the editors and the reviewers. Any product that may be evaluated in this article, or claim that may be made by its manufacturer, is not guaranteed or endorsed by the publisher.
